# A Novel Deep Learning Network and Its Application for Pulmonary Nodule Segmentation

**DOI:** 10.1155/2022/7124902

**Published:** 2022-05-17

**Authors:** Dechuan Lu, Junfeng Chu, Rongrong Zhao, Yuanpeng Zhang, Guangyu Tian

**Affiliations:** ^1^Cancer Center, Jiangdu People's Hospital, Yangzhou, Jiangsu, China; ^2^Department of Oncology, Jiangdu People's Hospital, Yangzhou, Jiangsu, China; ^3^Department of Medical Informatics, Nantong University, Nantong, Jiangsu, China

## Abstract

Pulmonary nodules are the early manifestation of lung cancer, which appear as circular shadow of no more than 3 cm on the computed tomography (CT) image. Accurate segmentation of the contours of pulmonary nodules can help doctors improve the efficiency of diagnosis. Deep learning has achieved great success in computer vision. In this study, we propose a novel network for pulmonary nodule segmentation from CT images based on U-NET. The proposed network has two merits: one is that it introduces dense connection to transfer and utilize features. Additionally, the problem of gradient disappearance can be avoided. The second is that it introduces a new loss function which is tolerance on the pixels near the borders of the nodule. Experimental results show that the proposed network at least achieves 1% improvement compared with other state-of-art networks in terms of different criteria.

## 1. Introduction

Lung cancer is the most deadly cancer in the world, which is characterized by high malignancy and indiscernibility [1]. Most cases of lung cancer are diagnosed when the cancer has already metastasized. Pulmonary nodules are the early manifestation of lung cancer, which appear as circular shadow of no more than 3 cm on the computed tomography (CT) image [1]. Pulmonary nodules in the early stage can be divided into solitary pulmonary nodules (SPNs), juxta-pleural nodules (JPNs), and ground glass opacity (GGO) [1]. Due to such complex diversity, the shape, size, density, location, and other characteristics of pulmonary nodules are quite different, which may lead to misdiagnosis. Therefore, accurate segmentation of the contours of pulmonary nodules can help doctors improve the efficiency of diagnosis.

In recent years, there are many segmentation methods for pulmonary nodules [2–5], which can be divided into two categories: the traditional unsupervised segmentation method and the machine learning-based segmentation method. Among the traditional unsupervised segmentation methods, the morphological method, threshold segmentation method, and clustering method are commonly used [6–10]. Although these methods are quick and simple, problems such as under-segmentation or over-segmentation still exist. Morphological methods can remove the edge burr of pulmonary nodules, but the parameters involved in the operation are not easy to control. The threshold segmentation method is not ideal in the segmentation of vascular adhesion pulmonary nodules. Sun et al. [11] used the maximum expectation algorithm and mean shift method to extract pulmonary nodules and achieved good results. However, the segmentation effect of this method was not ideal when the number of nodules was greater than or equal to 2. Although the gray threshold method proposed by Armato et al. [12] improves the segmentation accuracy, this method is time-consuming, limited, and inconvenient to use. Kanazawa et al. [13] used fuzzy clustering algorithm to extract lung and pulmonary vascular regions but lost 3D spatial feature information. Miwa et al. [14] proposed an algorithm called variable N-quoit filter for automatic recognition of pathological shadow candidates, which requires excessive manual operation and low automation.

Recently, deep learning-based methods have achieved great success in computer vision [15]. Deep learning can automatically extract features from training data and can produce fewer false judgments compared to traditional fine segmentation methods. In 2015, Long et al. [16] proposed fully convolutional network (FCN) to achieve end-to-end pixel-level prediction, which has become a trend in biomedical image segmentation. Researchers have also rapidly applied FCN to the field of medical imaging, and FCN has shown good performance in some high-intensity lesion segmentation. Based on FCN, Ronneberger et al. [17] proposed the U-NET for medical image segmentation, which is more suitable for biomedical image data processing with small data volume and can obtain better segmentation results. Due to U-NET's excellent performance in biomedical image segmentation tasks, in the following years, citations of the original U-NET literature proliferated. In addition, a number of improvements based on U-NET are also emerging. For example, Tong et al. [18] introduced the idea of residual network into U-NET to improve the segmentation accuracy of pulmonary nodules. Liu et al. [19] proposed a cascaded dual-path residual network segmentation method for pulmonary nodules, which was slightly more accurate than human experts. Zhong et al. [20] introduced the dense connection into U-NET which can not only strengthen the transmission and utilization of features but also avoid the vanishing gradient problem. Hou et al. [21] combined 3D-UNET and fully connected conditional random fields to improve the segmentation accuracy of pulmonary nodules. In 3D-UNET, the spatial and context information of pulmonary nodules was integrated to extract the different resolution characteristics. Moreover, in fully connected conditional random fields, the relationships between pixels were considered to optimize the previously rough segmentation with a step of resegmentation.

Although existing U-NET-based works have won great achievements for pulmonary nodule segmentation, there are still some existing shortcomings that should be further addressed.

1. The imbalance problem always exists in pulmonary nodule segmentation due to small size of pulmonary nodules. Therefore, how to choose a good loss function to overcome imbalance problems should be carefully considered.
2. Very discriminant features are very significant in pulmonary nodule segmentation. How to extract features from tiny targets is also very important.

In this study, based on U-NET, we propose a new network DENSE-UNET for pulmonary nodule segmentation. This new network enhances the transmission and utilization of the features, which can effectively alleviate the class imbalance problem and has a great improvement in the segmentation of small target regions such as pulmonary nodules. The main contributions of this paper are as follows:

1. A dense connection from DENSE-NET is introduced to U-NET to combine the features between the upper and lower convolutional layers. In view of the difficulty of feature extraction in some small target regions, the dense connection strategy can enhance the transmission and utilization of features in the network and solve the problem of gradient disappearance.
2. To avoid forcing the network to rigorously learn from an imprecise ground truth which often leads to over-fitting problems, we introduce a new loss function which is tolerant on the pixels near the borders of the nodule.

Different from U-NET, the proposed network uses dense connection to combine the features between the upper and lower convolutional layers. Additionally, a new loss function is used, which is tolerance on the pixels near the borders of the nodule.

The remaining sections are arranged as follows. In Section 2, we give the structure and loss function of the proposed DENSE-UNET. In Section 3, we report the experimental results. Lastly, we conclude the whole work and indicate our future works.

## 2. DENSE-UNET

### 2.1. Network Structure

In classical U-NET [17], the features extracted from each layer are usually learned only once, and there is a lack of connection between features of different layers. Therefore, the classical U-NET has low utilization for features, which affects the final segmentation accuracy. Unlike U-NET, DENSE-NET uses dense connection to combine the features of the current layer in the network with those of all previous layers and transmit the resulting features to all subsequent layers. In this cascading way, each layer in DENSE-NET can learn the features of the previous layers, which can not only strengthen the transmission of features by the network, so as to achieve feature reuse, but also alleviate the problem of gradient disappearance in the network. Suppose *o*_*l*_ is the output of the *l*-th layer in DENSE-NET, and thus we have

(1) $$\begin{array}{c} o_{l}=H_{l}\left(\left[o_{l-1},o_{l-2},\ldots ,o_{0}\right]\right), \end{array}$$

where *H*_*l*_() is a nonlinear function of the *l*-th layer and [*o*_*l*−1_, *o*_*l*−2_,…, *o*_0_] represents feature fusion of different layers.

**Figure 1:**
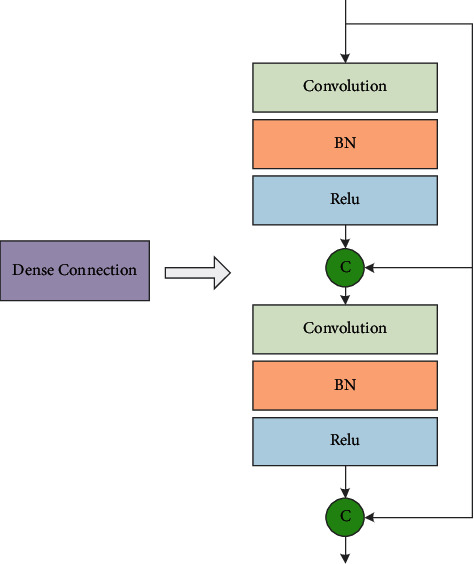
Structure of dense connection.

In this study, by virtue of the concept of dense connections from DENSE-NET, we design the structure of dense connection, as shown in Figure 1. We see that each dense connection module mainly contains two 3 × 3 convolution layers and two feature fusion operations. For the feature graph of the input dense connection module, after each convolution operation, the generated feature graph will be fused with the original feature graph to form a new feature graph, and finally the feature graph will be input to the next dense connection module.

In addition, a batch normalization (BN) layer and a rectified linear unit (Relu) activation layer are added behind each convolution layer to improve the performance of the network. The batch normalization layer is proposed to solve the problem that the training effect of network is easily affected by the initial data distribution and the model generalization ability is poor. The batch normalization layer is first used to normalize the input data as

(2) $$\begin{array}{c} \mu_{B}=\frac{1}{m}\sum_{i=1}^{m}x_{i},\\ \sigma_{B}=\frac{1}{m}\sum_{i=1}^{m}{\left(x_{i}-\mu_{B}\right)}^{2},\\ {\hat{x}}_{i}=\frac{x_{i}-\mu_{B}}{\sqrt{\sigma_{B}^{2}+\varepsilon }}, \end{array}$$

where *m* is the batch size, *σ*_*B*_ is the mean value, *σ*_*B*_^2^ is the deviation, and *ε* is a smoothing factor to avoid zero denominators.

The above operation changes the distribution of features that the network learns. In order to avoid the effect of network learning being affected by the change of feature distribution, the normalized data need to be transformed and reconstructed by

(3) $$\begin{array}{c} y_{i}=\gamma {\hat{x}}_{i}+\beta , \end{array}$$

where *γ* and *β* are learnable refactoring parameters. *y*_*i*_ is the output of batch normalized input data on the network.

The rectified linear unit activation layer is responsible for mapping the input to the output of the neuron in the neural network, which introduces nonlinear factors into the network, thus improving the nonlinear expression ability of the network. This layer is defined as

(4) $$\begin{array}{c} f\left(x\right)=\text{max}\left(0,x\right). \end{array}$$

**Figure 2:**
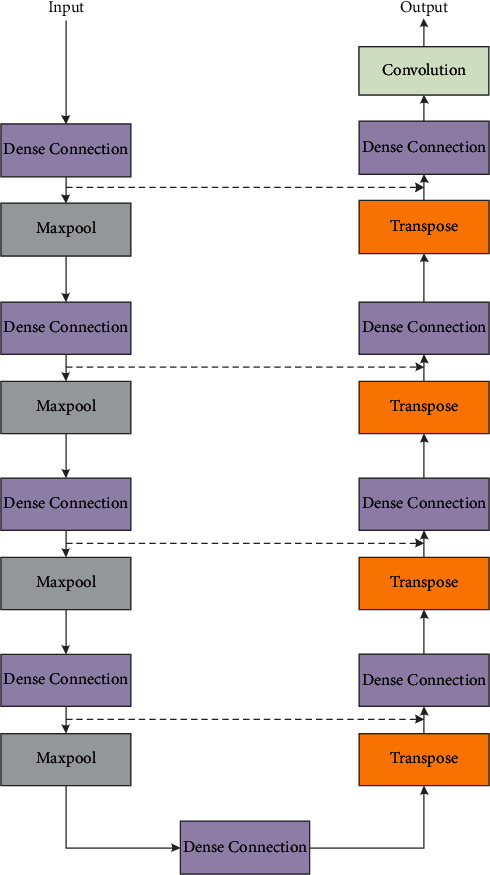
Structure of DENSE-UNET.

Based on the classical U-NET and combined with the dense connection module, we design an improved DENSE-UNET model, as shown in Figure 2. DENSE-UNET consists of an encoder, decoder, classifier, and skip connection. The encoder contains intense connection module and maximum pooling layer, where the dense connection module by convolution layer is used to extract image semantics, and the maximum pooling layer is used for the downsampling operation, which aims at reducing network computation and increasing the receptive field, so as to improve the robustness of image features. For the original input image, two convolution operations will be carried out through the dense connection module to obtain the 64 × 64 feature map, and then the size of the feature map is halved by pooling. Finally, after four times of convolution and pooling, the feature map of 4 × 4 size is obtained.

The decoder includes a dense connection module and a deconvolution layer, in which the deconvolution layer is used for the upsampling of the feature map to recover the resolution. After each deconvolution, the size of the feature map will be doubled, and finally the feature map with the same size as the original input image can be obtained. In addition, the encoder and the decoder are linked by a dense connection module.

The classifier consists of a 1 × 1 convolution layer and a sigmoid activation layer, where the 1 × 1 convolution layer is used to reduce the number of feature maps, and the sigmoid activation layer is used to determine the class of each pixel in the final feature map so as to output the final segmentation result. The skip connection combines the simple features with the deep features in the network so as to obtain more fine segmentation results.  Table 1 shows the parameter setting of each layer in DENSE-UNET.

### 2.2. Loss Function

In many segmentation networks, a common problem is arising, that is, precision of the boundaries of pulmonary nodules is often not available. This is caused not only by the loss function itself but also by the masks provided by the training dataset, which in many cases is not very accurate. Forcing the network to rigorously learn from an imprecise ground truth often leads to over-fitting problems. Therefore, inspired by the mean square error (MSE), we introduce a new loss function which is tolerant on the pixels near the borders of the nodule. To be specific, the proposed loss function measures the loss between one pixel and all the pixels around the corresponding one in the ground truth, inside a specific area. Then, it takes the minimum of those values. The radius of that area is Δ ≤ *f*(*d*_*no*  *d*_), where *d*_*no*  *d*_ represents the diameter of the nodule and *f* represents a function that is defined in (5). From (5), we see that *f* actually reflects the relationship between the diameter of nodules and the radius of the specific area.

(5) $$\begin{array}{c} f\left(d_{no\;d}\right)=\left\{\begin{array}{c} 0\text{ if}\frac{d_{no\;d}}{n}<64\\ 1\text{ if}\frac{d_{no\;d}}{n}<16\\ 2\text{otherwise } \end{array}\right., \end{array}$$

where *n* is the total number of elements of the image and the unit of distance is in pixels. Furthermore, it is coupled with an exponential preventing under-segmentation. That is to say, the proposed loss function is defined as

(6) $$\begin{array}{c} L\left(g_{i},\;p_{i}\right)=\frac{1}{n}\sum_{i=1}^{n}{\left(g_{i}-p_{i}\right)}^{2}\sigma^{\left(g_{i}-p_{i}\right)}, \end{array}$$

where *g*_*i*_ is the ground truth of the *i*-th pixel, *p*_*i*_ is the prediction result of the *i*-th pixel, and *σ* is a hyperparameter that is determined by users.

## 3. Experimental Results

### 3.1. Datasets

The training and validation sets are collected from the public dataset LIDC-IDRI which is sponsored and provided by the National Cancer Institute of America [22]. LIDC-IDRI contains CT images of 1018 patients. The nodule segmentation of each CT image is guided by 4 radiologists. The testing set is collected from the People's Hospital of Jiangdu which contains 100 patients.

In this study, we select 4000 CT images (slice < 3 mm) from LIDC-IDRI as training samples and 500 CT images (slice < 3 mm) from LIDC-IDRI as validation samples. We also select 200 CT images (slice < 3 mm) from Jiangdu People's Hospital as testing samples. The ground truth of the testing samples is obtained by manual segmentation.

**Figure 3:**
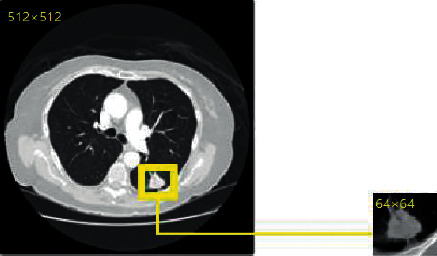
Example of original CT image reduction.

Since the sizes of pulmonary nodules are far less than those of the background, the imbalanced number of positive and negative samples will seriously affect the training process of the neural network and ultimately affect the segmentation performance of the network. Therefore, in this study, the original CT image samples containing pulmonary nodules are clipped to reduce the interference of other lung tissues on the experimental results. The size of the original CT image is 512 × 512. We cut the original CT image into an image with a size of 64 × 64, as shown in Figure 3.

### 3.2. Settings

To evaluate the segmentation performance of pulmonary nodules, the Dice similarity coefficient (Dice), precision, and recall were used as evaluation indexes, which are defined as follows:

(7) $$\begin{array}{c} \text{Dice}=\frac{2\sum_{i=1}^{N}g_{i}p_{i}}{\sum_{i=1}^{N}g_{i}+\sum_{i=1}^{N}p_{i}},\\ \text{precision}=\frac{\sum_{i=1}^{N}g_{i}p_{i}}{\sum_{i=1}^{N}p_{i}p_{i}},\\ \text{recall}=\frac{\sum_{i=1}^{N}g_{i}p_{i}}{\sum_{i=1}^{N}g_{i}g_{i}}, \end{array}$$

where *g*_*i*_ is the ground truth of the *i*-th pixel and *p*_*i*_ is the prediction result of the *i*-th pixel. From the above definitions, we see that Dice is used to measure the degree of similarity between the predicted results and the real results. Precision refers to the proportion of the total number of pixels correctly predicted as pulmonary nodules to the total number of pixels predicted as pulmonary nodules, and recall refers to the proportion of the total number of pixels correctly predicted as pulmonary nodules to the total number of pixels actually. The higher their value, the better the segmentation result.

To highlight the performance of our proposed network DENSE-UNET, FCN_32s [16], SegNet [23], U-NET [17], and U-NET with batch normalization (BN-U-NET) are introduced. All of these benchmarking networks follow the default settings. As for DENSE-UNET, Adam is adopted as the optimizer, the batch size is set to 32, the learning rate is set to 1e − 6, the momentum parameter is set to 0.8, and the number of training iterations is set to 50.

The experimental environment of this study is built based on the deep learning framework TensorFlow combined with Python. The computer configuration is as follows: the operating system is Windows 10, the processor is Intel Core i7-9700, the graphics card is NVIDIA RTX 2080TI of 11 GB memory size, and the system memory size is 32 GB.

### 3.3. Segmentation Results and Analysis

Table 2 shows the segmentation results of different networks on the testing set. From Table 2, we see that FCN_32s and SegNet perform worse than the other three kinds of models, and this is because FCN_32s and SegNet did not make full use of the extracted image features of each layer of the network. As for the features extracted by the encoder, FCN_32s and SegNet only carried out the upsampling to resize to the input image size, while ignoring the multiscale features on the space and the connection between the pixel location and classification. Therefore, FCN_32s and SegNet generate coarse segmentation results.

Unlike FCN_32s and SegNet, skip connection is introduced into U-NET, U-NET + BN, and DENSE-UNET based on the U-NET structure, which enables the network to combine the simple features of the shallow layer with the abstract features of the high layer. Since the number of high-quality medical images is small and the contrast between target region and background is low, it is difficult to extract features from them. Therefore, such kind of network is more suitable for medical image segmentation. By virtue of skip connection, simple features are used for accurate pixel positioning and abstract features are used for accurate pixel classification. That is to say, the two kinds of features are combined to help the network obtain more precise segmentation performance. In our experiments, our network has Dice of 0.7442, precision of 0.7551, and recall of 0.7254, which performs better than U-NET and BN-U-NET. This is because on the one hand, the improved DENSE-UNET not only used the skip connection modes but also fused the features extracted from the adjacent network layers. Such structure not only strengthened the transmission and utilization of features but also can alleviate gradient disappearance problems. Therefore, the improved DENSE-UNET has a stronger learning ability for the small or fuzzy boundary pulmonary nodules in the image. On the other hand, the improved DENSE-UNET can effectively unify the distribution of training data through batch normalization operation, thus improving the convergence speed of the network, improving the optimization performance of the network, and obtaining more accurate segmentation results.

## 4. Conclusion and Future Work

Accurate segmentation of pulmonary nodules is very important for the early diagnosis of lung cancer. Based on the U-NET structure, we proposed a novel network termed as DENSE-UNET to segment pulmonary nodules from CT images. The improved DENSE-UNET introduced the dense connection module between the convolutional layers, integrated the features of the upper and lower layers of the network, further strengthened the transmission and utilization of the features of the network, effectively improved the segmentation performance of the network, and solved the problem of gradient disappearance. At the same time, the improved mixed loss function is used to help the network optimize the samples that are difficult to learn stably and targeted, so as to solve the class imbalance problem faced by the network in the training process. The experimental data in this study are from the public database LIDC-IDRI and the Jiangdu People's Hospital. By comparing with the experimental results of FCN_32S, SEGNET, U-NET, and BN-U-NET, it is shown that the improved DENSE-UNET can effectively distinguish lung nodules from the background region, can achieve accurate segmentation of lung nodules, and has good segmentation performance.

However, the algorithm in this paper still has some limitations. As pulmonary nodules exist in multiple CT sections, the method in this paper only focuses on the lesion area in a single section and ignores the connection between adjacent section images, which inevitably leads to some misdetection or missed detection for small pulmonary nodules. Therefore, using the pixel connection between pulmonary nodules in adjacent sections to improve the segmentation accuracy will be the focus of the next research.

## Figures and Tables

**Table 1:** Parameters in each layer.

Layers	Size of feature map	Size/step of convolution
Input	64 × 64	—
Dense connection	64 × 64	[3 × 3 Conv-64] × 2
Max pooling	32 × 32	2 × 2/2
Dense connection	32 × 32	[3 × 3 Conv-96] × 2
Max pooling	16 × 16	2×2/2
Dense connection	16 × 16	[3 × 3 Conv-128] × 2
Max pooling	8 × 8	2 × 2/2
Dense connection	8 × 8	[3 × 3 Conv-256] × 2
Max pooling	4 × 4	2 × 2/2
Dense connection	4 × 4	[3 × 3 Conv-512] × 2
Max pooling	8 × 8	2 × 2/2
Dense connection	8 × 8	[3 × 3 Conv-256] × 2
Max pooling	16 × 16	2 × 2/2
Dense connection	16 × 16	[3 × 3 Conv-128] × 2
Max pooling	32 × 32	2 × 2/2
Dense connection	32 × 32	[3 × 3 Conv-96] × 2
Max pooling	64 × 64	2 × 2/2
Dense connection	64 × 64	[3 × 3 Conv-64] × 2
Max pooling	64 × 64	1 × 1 Conv

**Table 2:** Segmentation results of different networks in terms of Dice, precision, and recall.

Networks	Dice	Precision	Recall
FCN_32s	0.6885	0.7025	0.6781
SegNet	0.6944	0.7214	0.6841
U-NET	0.7211	0.7225	0.7234
BN-U-NET	0.7320	0.7454	0.7148
DENSE-UNET	**0.7442**	**0.7551**	**0.7254**

## Data Availability

The data are available on LIDC-IDRI which is sponsored and provided by the National Cancer Institute of America. The code can be accessed by sending e-mail to the corresponding author.
